# The abolition of user charges and the demand for ambulatory visits: evidence from the Czech Republic

**DOI:** 10.1186/s13561-016-0105-7

**Published:** 2016-07-15

**Authors:** Jana Votapkova, Pavlina Zilova

**Affiliations:** Institute of Economic Studies, Faculty of Social Sciences, Charles University, Opletalova 26, Prague, CZ-110 00 Czech Republic

**Keywords:** Co-payments, Outpatient care, Czech Republic, Natural experiment, Zero-inflated negative binomial model (ZINB)

## Abstract

This paper estimates the effect of the abolition of user charges for children’s outpatient care (30 CZK/1.2 EUR) in 2009 on the demand for ambulatory doctor visits in the Czech Republic. Because the reform applied only to children, we can employ the difference-in-differences approach, where children constitute a treatment group and adults serve as a control group. The dataset covers 1841 observations. Aside from the treatment effect, we control for a number of personal characteristics using micro-level data (European Union Statistics on Income and Living Conditions). Using the zero-inflated negative binomial model, we found no significant effect from the abolition of user charges on doctor visits, suggesting either that user charges are ineffective in the Czech environment or that their value was set too low. On the contrary, personal income, the number of household members and gender have a significant effect. A number of robustness checks using restricted samples confirm the results.

## Background

In many countries, governments have been increasing the rate of private participation in heath care expenses. The Czech Republic is not an exception. In 2005, general government expenditure amounted to 87.5 % of the overall spending on healthcare, but by 2009, this share had decreased to 83.6 % [9]. A number of reforms have been introduced in recent years, including the introduction of user charges for healthcare services, which started in January 2008 with the goal of reducing the unnecessary overuse of cost-free healthcare services by patients, thereby saving public resources. Nevertheless, in 2009 user charges for physician visits were abolished for children.

Three types of user charges were introduced in the Czech Republic in January 2008: CZK 30/1.2 EUR for physician visits during which a clinical examination was carried out; CZK 30/1.2 EUR for every item on a drug prescription; CZK 60/2.4 EUR for each day of inpatient care; and CZK 90/3.6 EUR for emergency service. This reform received extensive discussion. Advocates argued that user charges would have the desired effect and patients would decrease their overutilization of healthcare services because they would think twice about whether they needed to go to the doctor, i.e., the moral hazard would decrease significantly as suggested by [6], who addresses the theory of demand for health care. Feldstein [6] highlights that moral hazard results in the inefficient use of healthcare services and suggests that the introduction of co-payments is an effective way to reduce overutilization. Consequently, waiting times for physical examinations and planned surgeries would shorten, which should contribute to overall patient satisfaction with the particular service. In aggregate then, even this small amount of user charges should bring additional funds to the healthcare system.

To the contrary, opponents of the reform argued that in the Czech Republic (CR), the introduction of user charges would not have a significant effect on the demand for health-care services because this demand is not very price-elastic. Others claimed that user charges should at least not be applied to some vulnerable groups because their utilization of health-care services is appropriate, not excessive, and user charges could have a detrimental and inequitable effect on their health status as supported by [7, 15, 25] and [24]. As a result, in April 2009, user charges on physician visits were abolished for children up to 18 years of age, and a cap on copayments for the elderly (over 65) was decreased from CZK 5000 to CZK 2500.

Similar reforms introducing user charges have been tackled in other Visegrad countries. In 2007, Hungary instituted a 600 Forint/2.4 EUR user charge for a GP visit and 300 Forint/1.2 EUR charge for a specialist visit with a GP’s referral. A public referendum, however, abolished these a year later [2]. Slovakia instituted user charges in 2002, but a new government that took over in 2006 immediately abolished user charges [23].

The first studies to assess the effectiveness of user charges include [8, 16] and [17], who use data from a US social experiment - the Rand Health Insurance Experiment (HIE). All of these studies consistently find that in the short term, user charges that are too low do not reduce excessive care and user charges that are too high can result in avoiding necessary healthcare. These studies also show that for a person of average health and with average income, a reasonable level of user charges does not have a negative influence on health status. Saltman et al. [19], however, argue that these effects from cost-sharing arrangements may be valid only in the US and that studies performed in other countries could find different results.

As far as later studies are concerned, user charges on physician visits were found not to reduce demand for health-care services in South Korea [13] and in France [3]. Other studies find mixed results. In Japan [12], the effect of increased copayments for physician visits was found to be negative and statistically significant only for a two-year panel, but the effect was not clear for data acquired for longer periods. This phenomenon was interpreted as a transitory effect. In Belgium, [4] found negative effects from increased user charges on the demand for three types of physician services (GP office visits, GP home visits, specialist visits); however, in disaggregation, the effect was insignificant for men visiting GP offices and for women visiting a specialist. In Germany, [29] found that an increase in user charges for drug prescriptions fulfilled its purpose of reducing the number of outpatient doctor visits. However, as [1] and [22] discovered, further German reforms that introduced user charges for the first doctor visit in each quarter in 2004 failed to reduce the number of physician visits. Significance and the effect of user charges thus depend on the amount, frequency of payment, type and characteristics of each country.

The effect of user charges in the Czech Republic has been estimated only by [30] and [11] to date. Zapal [30] estimated the effect of user charges on the number of children’s physician visits in the Czech Republic, proxying the number of doctor visits by the number of drug prescriptions under the assumption that there is a fixed probability of generating prescriptions during a doctor visit. The author detects a positive and significant effect from user charges only if March 2009 (one month before the reform) is used as a pre-reform period, i.e., there is only a timing effect because some visits (e.g., preventive care) might have been postponed, resulting in fewer visits prior to the reform and more visits after it. In a natural experiment, [11] estimated the effect of user charges on healthcare utilization among the Czech population above 50 years of age using data from the Survey of Health, Aging and Retirement in Europe (SHARE). She found a significant decrease in ambulatory doctor visits after the introduction of user charges but an insignificant effect from the reform on the amount of hospital care provided.

Except for [13], who conducted a conditional-on-use analysis, all of the cited research papers investigate the effect of user charges on doctor visits using the difference-in-differences (DiD) methodology. In the Czech environment, [30] takes advantage of the co-payment exemption for children introduced in 2009 and uses children’s drug consumption as a treatment group and drug consumption among adults as a control group. He employs Ordinary Least Squares (OLS). Kalousova [11] assumes that the Czech Republic and Poland would experience identical trends were it not for the introduction of user charges in the Czech Republic. In her experiment, the Czech population constitutes a treatment group and the Polish population serves as control group. She employs logistic regression and binomial models.

We will contribute to this stream of research and analyze the effects of user charges on the number of outpatient visits in the Czech Republic. We will estimate the effect of the 2009 abolition of user charges for children and thus find whether the introduction of regulatory fees in the Czech Republic reduced the overutilization of outpatient healthcare services. In our natural experiment, we will assume elastic healthcare demand for both children and adults, which is supported by [20].

As opposed to [11], both the treatment and control groups will be subsets of the Czech population, which allows us to relax the assumption about identical trends for the Czech Republic and Poland, which we consider to be quite restricting given the different institutions in the respective countries. In addition, we will not limit ourselves to the elderly (50+), as opposed to [11]. In our experiment, 281 children will constitute a treatment group, whereas the rest of the population (1,560 adults) will serve as a control group. We will take advantage of the fact that the dividing line (18 years) is exogeneously (administratively) given and assume that the trend is the same for both groups. The appropriateness of the identical trend assumption will be tested in a robustness check in which we restrict the control group (18+) to the age group 18–26. As opposed to [30], we will use micro-level data on the number of doctor visits made by individuals during the 12 previous months, as obtained from the European Union Statistics on Income and Living Conditions (EU-SILC survey). Moreover, a longer time period allows us to eliminate the timing effect from the postponed utilization of health-care services observed by [30]. Due to the distributional properties of the dependent variable (number of doctor visits), we will avoid OLS, as opposed to [30], and logistic regression and the binomial model, as opposed to [11], and use the zero-inflated negative binomial (ZINB) model, which provides a better fit to the data due to skewness and a large number of zero doctor visits in the dataset. Consistent with [30] and as opposed to [11], our analysis covers the area of the city of Prague only because co-payment arrangements are different outside of Prague. Furthermore, there is believed to be hardly any patient spillover from Prague to other regions because (i) the quality of healthcare services is generally perceived to be same or better in Prague than in the rest of the Czech Republic and (ii) the cost of travel outside Prague is higher than 30 CZK/1.2 EUR.

Our research questions are as follows: (1) Did the abolition of outpatient user charges have a significant effect on the demand for outpatient doctor visits? (2) How do individual characteristics such as sex and income affect the demand for ambulatory healthcare services?

If the number of children physician visits increased after the abolition of user charges in 2009, the introduction of regulatory fees in 2008 would have been effective in reducing demand. We however found an insignificant effect, i.e., the number of children’s outpatient visits (treatment group) did not significantly change after the abolition of regulatory charges. The results suggest that the reform either did not have an effect or that the user charges which the reform abolished were too small to have an effect on the behavior of patients. We further discovered that the probability of visiting a doctor increases for women and decreases with personal income and the number of household members. Note that we are limited by the sample size of 1,841 individuals, which includes 15 % of children (members of the treatment group). If more data was available, the estimates would by definition be more accurate due to lower variance. The results are nevertheless not biased by the disproportionate number of members in the treatment and control groups which is confirmed by a number of robustness checks. Eight robustness checks were carried out with alternative control groups and estimation methods.

The paper is organized as follows: “Data and methods” Section introduces the dataset and explains the theoretical underpinnings, “Results” Section presents and discusses the results of the analysis, and “Discussion and conclusion” Section concludes and provides motivation for further research.

## Data and methods

### Data

The data come from the Czech Statistical Office (CZSO), the EU-SILC survey, which is an annual survey of household income and living conditions and includes data on health related variables, such as the number of doctor visits during the 12 previous months, and respondent characteristics associated with a tendency toward health-care utilization (age, sex, educational level, marital status, employment status, household income per year, number of children in a household, etc.) [5]. The respondents above 16 years of age answer the questions themselves, while legal guardians answered on behalf of children younger than 16. These children are included in our sample because answers on behalf of children are not considered to be a problem for the purpose of our analysis.

Our sample covers only two interview years (2009, 2010) because information regarding the utilization of health services in the past 12 months was not included in earlier surveys and because data for later years are not available. We restricted the sample such that all answers refer fully either to the period before the reform, which took place in April 2009, or after it to avoid bias in the results due to overlap. Because we know the exact day of each interview, we limited the sample to two interview periods: (1) From February to March 2009, i.e., responses regarding the number of doctor visits in the past 12 months effectively refer to the period between February 2008–March 2009 when user charges were obligatory for all, and (2) From April to May 2010, i.e., responses refer to healthcare utilization between April 2009–May 2010 when children were exempted from user charges.^1^

From the overall sample, we excluded all people living outside of Prague because the data for the rest of the country could be contaminated by the fact that other regional governments except Prague reimbursed adults for co-payments in all regional hospitals (but not others) during the observed period. Being unable to distinguish whether the patient took advantage of reimbursement could influence our estimated results. Moreover, we suppose that people living in Prague always go to a doctor in Prague because all types of primary and secondary care are represented there and the capital is generally perceived by the public to provide higher quality healthcare. Additionally, if a Prague citizen wanted to avoid paying user charges by going to a doctor outside Prague, his travel costs would exceed CZK 30/1.2 EUR. Lastly, we truncated our sample at 20 visits. The final dataset covers 97.3 % of the non-truncated set and includes 1,841 individuals - 281 children and 1,560 adults. The proportion of children and adults is consistent with the overall sample.

#### Dependent variable

**Fig. 1:**
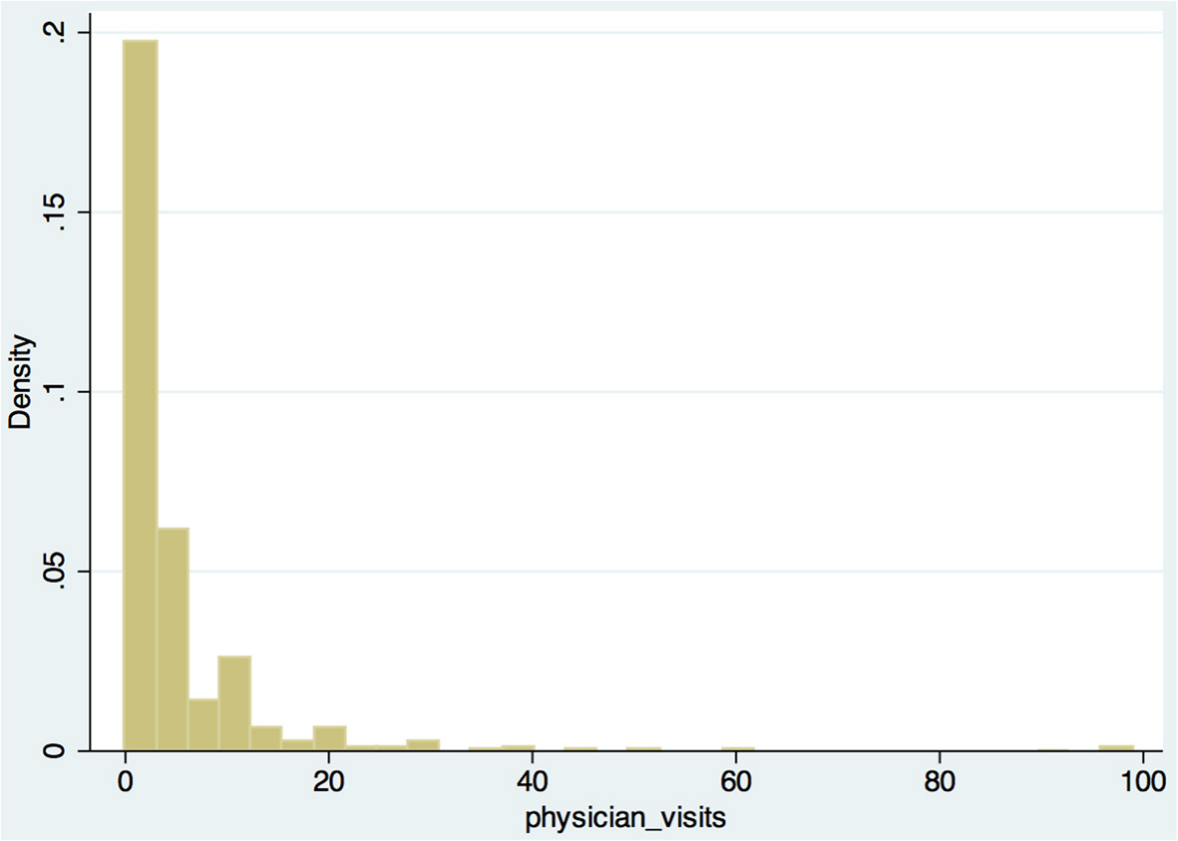
Frequency distribution - visits

The dependent variable *visits* denotes the number of physician visits (generalist or specialist with and without a referral) made by an individual during the 12 previous months. The frequency distribution in Fig. 1 indicates a rapidly decreasing tail and suggests that the distribution is not normal.

The maximum number of visits is 99; however, only 2.7 % of respondents exceed 20 visits. The density of observations above 20 visits is also extremely low (Fig. 1). We thus truncated the sample at 20 visits. We suppose that some of the high values may be results of measurement errors; others may be brought about by the elderly (over 65), who visit doctors more often. Truncation at 20 visits eliminates these erroneous observations as well as removes a potential bias stemming from a decreased cap on out-of-pocket contributions for the elderly introduced in 2009. In other words, we assume that a greater number of visits (made mostly by the elderly and chronically ill) is refunded because these individuals are also expected to pay out-of-pocket at the pharmacy, which adds to the copayment limit as well.

#### Independent variables

Aside from the interaction term, which captures the effect of the reform, there are other independent variables that are likely to influence the number of outpatient visits and reflect characteristic differences among patients.

The variable *female* is a dummy taking on the value of 1 for a female and 0 for a male. The mean (Table 1) reveals that our sample contains approximately the same number of men and women. We expect a positive effect from this variable because women have a larger number of comorbidities than men, which is proved for instance by [18] and [28].

**Table 1:** Summary statistics

Variable	Mean	Std. Dev.	Min.	Max.	Median
Female	0.528	0.499	0	1	1
Visits	3.539	4.533	0	20	2
Members	2.806	1.211	1	7	3
Reform	0.382	0.486	0	1	0
Dummy_child	0.153	0.36	0	1	0
Interaction	0.062	0.241	0	1	0
Log_income_memb	12.002	0.494	10.375	14.271	11.948

The variable *log_income_memb* denotes the net disposable income of a household per year (as defined by the EU) divided by the number of household members. This variable enters the regressions in natural logarithms because in natural units, it takes on a wide range of values. The impact of this variable may be two-fold: (a) With increasing income, the number of doctor visits may grow because money spent on health-care expenses becomes unimportant. This effect is supported for example by [26]; (b) With increasing income, the number of doctor visits decreases because people have a better lifestyle (they buy better food, shoes, mattresses, etc.) as well as health status. However, a low number of doctor visits for high income individuals may also be caused by the high opportunity costs of going to the doctor and not working, which is supported by [27], who found a higher use of GP care among lower-income groups. The final effect depends on which of these two effects outweighs the other.

The number of household members (*members*) takes on values from 1 to 7. We include this variable in the analysis based on [21], who found a significant effect from family size on health care utilization. Through the number of household members, we also try to capture relevant household characteristics to minimize dependence in the error term because with the available data, we cannot cluster members of the same family and capture their fixed attributes. Because there are 3 members in a median family in our sample (Table 1), the dependence is not expected to cause trouble in the analysis.^2^

We suppose a negative influence from this variable because increasing the number of household members may represent higher opportunity costs for the primary care-taker (e.g., to find baby-sitting) or tighter budget constraints for the family as a whole.^3^

Summary statistics for all variables are provided in Table 1. A correlation matrix can be provided upon request by the authors.

### Method

#### Difference-in-differences approach

The abolition of user charges for children’s outpatient visits in 2009 constitutes a natural experiment with children’s physician visits being the treatment group and physician visits for the rest of the population being the control group.

The DiD approach is based on a comparison of the average change in physician visits for children before and after the reform with the average change in physician visits for the adults. If we compared pre-reform and post-reform periods for the treatment group only, the results could be contaminated by trends that are unrelated to the reform. A parallel trend assumption for both groups must hold, however, to allow general conclusions to be drawn from the analysis. In our case, we assume that without the reform, the development of the trend in both the group of children and the adults would be the same, and thus, we could draw a conclusion regarding the effect of the reform for the entire population and not just the population of children. The appropriateness of this assumption will be tested in a robustness check using a restricted control group.

To find the effects of the reform, we estimate a model of the form:

1 $$\begin{array}{*{20}l} \text{visits}_{i}&=\beta_{0}+\beta_{1}\text{reform}_{i}+\beta_{2}\text{dummy\_child}_{i}\\ &\quad+\beta_{3}\text{interaction}_{i}+\beta_{4}\text{female}\\ &\quad+\beta_{5}\text{members}+\beta_{6}\text{log\_income\_memb}+\varepsilon_{i} \end{array} $$

where *i*∈1,…*N* denotes individuals. The variable *visits* reflects the number of doctor visits for person *i*. *Reform* is a dummy variable representing the period after the reform. The variable *dummy_child* is a dummy variable that takes the value of 1 for respondents younger than 18 and 0 otherwise. In other words, this variable denotes the treatment group, i.e., the group on which the reform had an effect. The *interaction* term equals *reform*×*dummy*_*child* and takes the value 1 for children (members of the treatment group) and the period after the reform. The variable *female* takes the value of 1 if the respondent is a woman. The variable *members* denotes members of the household, and the variable *income_memb* represents the personal income of individual *i*. The parameter *ε*_*i*_ is the error term.

We are particularly interested in the estimate of *β*_3_ because it gives us the net treatment effect, which measures the change in physician visits for a child caused by the abolition of user charges – it is the DiD estimator. If positive, the number of doctor visits for children rises relative to the number of visits for adults.

#### Zero-inflated negative binomial model

Because the distribution of the dependent variable *visits* is skewed and contains a large proportion of zeros, we use a count data model, specifically the zero-inflated negative binomial model (ZINB).

The ZINB model consists of two submodels because it assumes that zero values for the dependent variable are generated from two different processes [14]: (i) A respondent was not ill over the year and therefore did not visit a doctor and (ii) a respondent was ill, but still did not visit a doctor.

The first submodel is negative binomial and models the count process, i.e., how often respondents visit a physician. The dependent variable takes the value 0–20. The second process is modeled by a logit model for binary data to capture the probability that the respondent was ill but still did not visit a doctor. The dependent variable is a latent (unobserved) variable taking the value of 0 if the particular zero number of visits is likely to be caused by the fact that the respondent did not go to the doctor because he was healthy and taking the value of 1 if the zero number of visits is likely to be caused by the avoidance of healthcare even if sick.

## Results

We estimate Eq. 1. The estimation results are provided in Table 2.^4^

**Table 2:** Zero-inflated negative binomial model

	Number of obs = 1841	
	Non-zero obs = 1176	
	Zero obs = 665	
	LR *χ* ^2^(6) = 79.38	
	Prob >*χ* ^2^ = 0.0000	
	Visits	Coef.
Count process = neg. binomial		
	reform	0.010
	dummy_child	–0.242
	interaction	–0.497
	female	0.190***
	members	–0.132***
	log_income_memb	–0.337***
	_cons	5.840***
Inflation model = logit		
	reform	1.232***
	dummy_child	4.377***
	interaction	–0.588
	female	–1.233***
	members	0.502***
	log_income_memb	0.261
	_cons	–6.292***
	ln *α*	–0.451
	*α*	0.637

Likelihood-ratio test of *α* = 0: $\bar {\chi }^{2}$(01) = 1393.26 Pr$\geq \bar {\chi }^{2}$ = 0.0000

Vuong test of zinb vs. standard negative binomial: z = 6.91 Pr >z = 0.0000

Notes: * *p*<=0.1, ** *p*<=0.05, *** *p*<=0.01

In order to interpret coefficients of the log–linear model, exponentiating is necessary; exp(coeff)

The estimated coefficient of the interaction term, which denotes the treatment effect, i.e., the effect of the reform that abolished user charges for children, is insignificant in both parts of Table 2. This analysis thus reveals that the reform did not significantly change the utilization of doctor visits consistent with, for example, [13] and [3]. Specifically, the effect of the abolition of user charges for children was found not to play a role either in the first part of Table 2, which models how many times respondents visit a doctor, or in the second part of Table 2, which estimates the odds of not going to the doctor even when sick. In other words, the probability of avoiding healthcare if sick did not change after the reform; the reform also did not change the frequency of doctor visits.

If the reform had an effect, the coefficient of the interaction term would have been significantly positive in the first part of Table 2 and significantly negative in the second part of Table 2 because one would expect the number of doctor visits to increase and the odds of avoiding healthcare even if sick to decrease as a result of the abolition of user charges.

The first part of Table 2 further suggests that women are expected to visit the doctor 1.21 more times than men and a one-unit increase in the logarithm of personal income decreases the expected number of visits to the doctor by 0.71. These results confirm the findings of [18] and [28]. Also, the more household members there are, the less they are expected to visit a doctor, consistent with our assumptions. However, the specific reason for this result is yet to be clarified. We suppose that larger families may have a tighter budget constraint or the primary care-taker in larger families may face higher opportunity costs in terms of finding, e.g., baby-sitting.

The signs of all the coefficients for personal characteristics in the second part of Table 2 are opposite (if significant) to the signs of those in the first part of this table because each answers the question from a different perspective. The results in both parts of Table 2 thus nicely complement each other. The second part of Table 2 suggests that if a woman did not visit a doctor during last 12 months, it was more likely caused by the fact that she was healthy and hence did not need to visit a doctor than by the fact that she would avoid healthcare if sick. If members of larger families tend to go less frequently to the doctor (first part of the table), then they also more likely avoid seeing a doctor if sick. Income was found not to play a role in determining whether a respondent is likely or not to avoid healthcare services if sick.

### Robustness checks

We conducted four robustness checks to test the appropriateness of the assumptions applied to the primary analysis, including the parallel trend assumption^5^. As the number of members in the control group of each of the four robustness checks decreases relative to the main analysis, we check also for a potential bias resulting from a different size of the treatment and control groups in the main analysis.^6^

Additionally, all analyses, i.e., the full sample and the robustness checks, were re-estimated using a multinomial logit regression model (MNL), which estimates the probability of a change in the number of physician visits after April 2009. All results are consistent with those of the ZINB model and are available upon request from the authors.

#### Children vs. adults aged 18 to 26

The first robustness check is the core test of the parallel trend assumption, i.e., of the assumption that trends for the group of children and adults included in the analysis would be the same were it not for the reform applied to children. The control group covers only people aged 18 to 26 based on the assumption that the behavior of children and young people close to the administratively set borderline of adulthood should be more similar than that of children and older adults.

The results are provided in column 3 of Table 3 and reveal an insignificant effect from the interaction term in either part of the table, consistent with the primary analysis. Thus, we prove that the reform did not influence either the number of doctor visits or the probability of avoiding healthcare if sick. However in this analysis, no personal characteristics considered significantly influenced the dependent variable except that sex and the number of household members in the model estimate the probability of avoiding healthcare if sick. The magnitude of the effects of these two variables is weaker than in the primary analysis, but the directions of these effects are consistent. Specifically, being a woman decreases the probability of not going to the doctor even if sick by 0.38 and living in larger households increases it by 1.51, all else being equal.

**Table 3:** Zero-inflated negative binomial model: robustness checks

(1)	(2)	(3)	(4)	(5)	(6)
Control group		Adults aged 18 to 26	Childless adults	Adults without the elderly	Employed adults up to 65 years
		Number of obs = 494	Number of obs = 1243	Number of obs = 1471	Number of obs = 1117
		Non-zero obs = 130	Non-zero obs = 735	Non-zero obs = 837	Non-zero obs = 579
		Zero obs = 364	Zero obs = 508	Zero obs = 634	Zero obs = 538
		LR *χ* ^2^(6) = 3.60	LR *χ* ^2^(6) = 103.38	LR *χ* ^2^(6) = 38.56	LR *χ* ^2^(6) = 20.64
		Prob >*χ* ^2^ = 0.7301	Prob >*χ* ^2^ = 0.0000	Prob >*χ* ^2^ = 0.0000	Prob >*χ* ^2^ = 0.0021
Count process					
	reform	–0.249	–0.015	0.034	0.109
	dummy_child	0.172	–0.023	–0.072	0.133
	interaction	-0.659	–0.246	–0.583	–0.851
	female	–0.116	0.144**	0.368***	0.355***
	members	–0.029	–0.265***	–0.059*	0.016
	log_income_memb	–0.158	–0.461***	–0.202***	–0.003
	_cons	3.142	7.608***	3.675***	0.872
Logit					
	reform	0.725	0.807***	1.617***	1.605***
	dummy_child	3.284***	3.415***	4.765***	5.042***
	interaction	–0.233	–0.106	–0.988	–1.020
	female	–0.967***	–0.881***	–1.334***	–1.462***
	members	0.414**	0.660***	0.471***	0.333**
	log_income_memb	0.299	0.246	0.115	0.122
	_cons	–5.532	–5.895**	–4.809	–4.567
	ln *α*	–0.443	–0.757	–0.219	–0.242
	*α*	0.642	0.469	0.804	0.785

Notes: * *p*<=0.1, ** *p*<=0.05, *** *p*<=0.01

In order to interpret coefficients of the log–linear model, exponentiating is necessary; exp(coeff)

The insignificant effect of the remaining environmental variables is not a surprise considering that the two groups are assumed to be even more alike than the treatment and control groups of the primary analysis. We thus proved that the parallel trend assumption of the primary analysis holds even for the group of children and the rest of the population.

#### Children vs. childless adults

In the second robustness check, only childless adults are included in the control group based on the assumption that parents may be partly influenced by the reform as well: because when they no longer pay for their children’s visits, they may change their own decisions concerning physician visits for which they make co-payments. In other words, we check whether the behavior of parents and children is independent of each other.

The results, which are provided in column 4 of Table 3, are consistent with the primary analysis and reveal both the insignificance of the interaction term and the strong significance and identical direction of the effects of the environmental variables as before, suggesting that parents’ decisions regarding the number of their own doctor visits are independent of those made on behalf of their children.

#### Children vs. adults without the elderly

Although we already restricted our dependent variable to 20 visits in the primary analysis, assuming that the elderly go to the doctor more often, in the third robustness check, we further restrict the control group to the adults without the elderly (over 65) based on the fear that the results of the primary analysis may still be slightly biased due to the decreased cap on co-payments for the elderly introduced in 2009. The reason is that there are not only copayments for doctor visits, but also copayments for inpatient care and all out-of-pocket payments in the pharmacy that sum up to the limit. Once the limit is exceeded, the patient is eligible for reimbursement regardless of the number of doctor visits. Thus, there are only people aged 18–64 included in the control group.

The results provided in column 5 of Table 3 are consistent with our previous estimates. The coefficients of the interaction term in both parts of the table are statistically insignificant. Thus, we verified that the abolition of user charges did not have a significant effect on the number of doctor visits even if the elderly were excluded from the control group nor did it affect the probability of avoiding healthcare if sick. The results of the primary analysis are thus by no means distorted by the decreased copayment cap applied to the elderly. It is believed that the decreased protective limit may have rather significantly influenced the number of drug prescriptions or the utilization of health-care services above 20 outpatient visits.

All individual characteristics are jointly significant, which is consistent with the results of our primary analysis. There is only a slight decrease in the significance of the variable *members* from a 1 % significance level to a 10 % level.

If we consider that the elderly live more often in smaller households, the variable representing the number of household members then naturally plays a more important role in the primary analysis when the elderly are included. Put differently, the elderly are believed to be responsible for the stronger significance of this variable in the primary analysis.

#### Children vs. employed adults up to 65 years

In the fourth robustness check, there are only employed adults younger than 65 years in the control group based on the assumption that the employed are less affected by worse labor market conditions after the wake of the economic crisis at the end of 2008.

The results provided in column 6 of Table 3 are consistent with the primary analysis in terms of the treatment effect. Thus, we verified that the insignificant effect of the abolition of user charges is robust even to the joint exclusion of the unemployed and the elderly from the control group. Labor market conditions are perceived to work through the income variable, which also became insignificant in the first part of the table. Thus, if only the employed are included in the control group, the decision as to how many times to go to the doctor and whether to avoid healthcare if sick is not influenced by income. However, even this analysis proved that women go to the doctor more often and are less likely to avoid healthcare if sick. In addition, the number of household members influences the probability of not going to the doctor even when sick, increasing it by 1.4; the number of doctor visits is not influenced by the number of household members.

## Discussion and conclusion

This paper investigates the effect of the abolition of user charges on the demand for ambulatory doctor visits. It analyzes the EU-SILC micro-level data from the 2009 and 2010 surveys. The reform is a natural experiment in which children constitute a treatment group and the rest of the population serves as a control group. The natural experiment took place effectively in the city of Prague. Prague is the capital and the largest city in the Czech Republic; many specialized medical centers are situated there, including 5 out of 11 teaching hospitals (as of 2009). We abstract from any possible spillover effect because (i) no regional substitute exists for specialized care provided in Prague and (ii) regional substitutes for regular care are significantly more expensive due to considerable travel costs. In other words, it is quite common to obtain specialized treatment in Prague even if residing somewhere else, but hardly any Prague citizen would use a hospital outside Prague to avoid the user fees.

A zero-inflated negative binomial model was used as the estimation method. The model expresses the probability of visiting a doctor as a combination of two submodels, assuming that the zero number of doctor visits is generated by two different processes: (i) a respondent was not ill and therefore did not visit a doctor (but would visit if sick), and (ii) a respondent was ill, but still did not visit a doctor.

Our results show an insignificant effect from the abolition of user charges on the number of doctor visits, consistent with a number of previous studies, e.g., [3, 13, 30], etc. These results suggest that user charges for ambulatory doctor visits are either ineffective in reducing the overuse of healthcare services in the Czech environment or that their value was just set too low.

These results are not expected to be significantly distorted by a temporary effect, as found by [30]. Our data are collected over a period 12 times longer, i.e., we assess a period of one year - not one month, in which the people could indeed postpone the consumption of healthcare from the pre-reform period to the post-reform period to avoid user charges. As we expect both temporary and long-term effects to be non-negative, the resulting observed effect, which is their sum, is also expected to be non-negative if significant. However, the observed effect is insignificant here. Thus, if we subtract the temporary effect, the long-term effect must also be insignificant. By the same token, even if the discovered effect was deemed to be just temporary, i.e., one believes that people postpone healthcare consumption for a year to avoid user charges, the long-term effect would be even less significant than the observed one. Thus, a longer time period of observations, even if available, would not enhance the analysis in terms of the direction and significance of the effect.

Sex, personal income and the number of household members all proved to have a significant effect on the demand for outpatient care - being a woman increases the number of doctor visits, and the number of household members and personal income decrease it, suggesting that richer people have considerable opportunity costs from visiting a doctor. The finding that the bigger the household is, the less its members visit the doctor may also be explained by opportunity costs for both the primary care-taker and the bread-winner. Additionally, larger families are likely to face tighter budget constraints than smaller families. The specific reason for the direction of this effect is left as a motivation for further research.

The results further reveal that the odds of avoiding healthcare even if sick do not significantly change after the abolition of co-payments. The probability of not going to the doctor even if sick increases with the number of household members and decreases when the respondent is a woman.

The insignificant effect from the abolition of co-payments on the number of outpatient doctor visits proved to be robust to alternative control groups. Only minor changes in the effect of environmental variables in the alternative models were recognized.

Specifically, we tested that the control group of adults and the treatment group of children would experience the same trend were it not for the abolition of user charges for children in 2009. For this purpose, we restricted the control group to individuals aged 18–26 who were compared against children (0–18). The results were consistent with the primary analysis, confirming the accuracy of the parallel trend assumption. Second, we took childless adults as a control group and found that the behavior of the adults regarding their doctor visits and the doctor visits of their children are independent. Third, we tested for the presence of bias resulting from the decreased cap on co-payments from the elderly, which was also introduced in 2009. Restricting the control group to the adults without elderly, we rejected the presence of this type of bias in our primary analysis. Fourth, we tested whether deteriorated labor market conditions due to the financial crisis played any role. When excluding the elderly and unemployed jointly from the control group, we found no significant effect from labor market income. Fifth, the different numbers of observations in the control groups of the alternative models rejected a potential bias resulting from a different proportion of observations in the control and treatment groups in the main analysis.

Our primary results for a sample of the entire Czech population differ from the result reached by [11], who analyzed only the elderly, who may behave differently compared to the rest of the population. We accounted for the extreme behavior of the elderly by (i) truncating the dependent variable to 20 visits and (ii) conducting a robustness check excluding the elderly.

Still, the results of our analysis should be interpreted with limitations and could be extended in a number of ways. First, we assessed the effect of a small price change, which cannot predict how people respond to bigger price changes. Second, due to data availability, only 1841 observations were analyzed. A larger dataset would, by definition, decrease variance of the estimates making them more accurate. Third, the time period examined covered only two years of observations. A longer time period would by no means enhance the analysis in terms of the effect of the reform because the strongest response to the abolition of user charges would be right after its implementation. If data for additional years were available in the panel structure, we may however obtain additional information regarding fixed individual and household characteristics. This possibility also serves as a motivation for further research.

When conducting this analysis, we could not distinguish between emergency and ordinary visits due to data availability. If we are able to analyze such disaggregated data, we would additionally discover whether the people in the Czech Republic are sensitive in terms of the structure of user charges. In other words, we may find that it pays for some to wait a day or two before they go to a doctor. Considering that CZK 90/3.6 EUR for an emergency visit is three times the amount of the user charge for an ordinary ambulatory visit, one may find a more profound effect from the reform in this situation. At the same time, our present results are not assumed to suffer from large distortions because emergency ambulatory visits are significantly less frequent than ordinary visits, accounting for only 4 % of all ambulatory visits in 2009 [10]. This type of additional analysis is a motivation for further research.

Regardless, the user charge of 30 CZK/1.2 EUR for an ambulatory visit was found to be ineffective in the Czech environment. The task for a policy-maker and for further research is thus to discover what, if any, the appropriate amount and structure of user charges would need to be to decrease overuse of cost-free healthcare services in the Czech Republic.

## Endnotes

^1^ Interviews take place only between February–May each year.

^2^ Autocorrelation in the error term was rejected by a bootstrap analysis with 50, 500 and 1,000 replications. Results are available upon request from the authors.

^3^ The variable number of children in the household was also tested, but the number of household members was deemed to be more appropriate because families may take care of children, elderly parents, or both.

^4^ A significant ln*α* suggests overdispersion, which proves that the ZINB is appropriate. Moreover, the LR test of Vuong, which compares the ZINB model to the standard NB model, indicates that the ZINB model should be preferred to the NB regression model even at a 1 % significance level. Moreover, the likelihood-ratio statistic of 79.38, which has an *χ*^2^ distribution, reveals that the full model fits significantly better than an empty model.

^5^ An alternative test to check the parallel trend assumption is to conduct a placebo analysis, which was however impossible due to the setting of the reform and data availability.

^6^ Number of treatment group observations = number of observations - 281.
